# Early Career Psychiatrists in Europe During COVID-19 Outbreak: Results of The EPA ECPC-EFPT Cross-Sectional Survey

**DOI:** 10.1192/j.eurpsy.2022.202

**Published:** 2022-09-01

**Authors:** T. Gondek

**Affiliations:** European Psychiatric Association, Early Career Psychiatrists Committee, Wrocław, Poland

## Abstract

**Abstract Body:**

The COVID-19 pandemic has affected the lives and work of Early Career Psychiatrists (ECPs). Some had to revamp their professional life, start using telepsychiatry without prior training, change their workplace during the pandemic, or were quarantined. Others were not able to complete their training or take obligatory courses as planned. The aim of the study was to understand the impact of the pandemic on education and professional development, working conditions and wellbeing of ECPs, as well as their attitude to telepsychiatry. The anonymous, 24-question cross-sectional survey was conducted by the European Psychiatric Association Early Career Psychiatrists Committee (EPA ECPC) and the Task Force on Meetings and Associations with the collaboration and support of the European Federation of Psychiatric Trainees (EFPT). 517 participants from 39 different countries (member states of the Council of Europe) have been included in the analysis. Statistical analyses have been performed. Final results will be presented during the symposium. Identifying the impact of the COVID-19 pandemic on ECPs will help us better prepare for similar events in the future, equip them with the necessary skills and provide them with the right support.

**Disclosure:**

No significant relationships.

